# Effect of Functionally Graded Material on the Dynamic Stability of Three-Layered Annular Plates

**DOI:** 10.3390/ma19020256

**Published:** 2026-01-08

**Authors:** Dorota Pawlus

**Affiliations:** Faculty of Mechanical Engineering and Computer Science, University of Bielsko-Biała, 43-309 Bielsko-Biała, Poland; dpawlus@ubb.edu.pl

**Keywords:** three-layered annular plate, composite plate structure, functionally graded material facings, dynamic stability, finite difference method, finite element method

## Abstract

This study considers the dynamic stability of a three-layered annular plate, whose facings are made of functionally graded material in the radial direction. The plate is subjected to linearly increasing in-plane forces applied at either the inner or outer edge. The effect of the heterogeneity of the plate-facing material on the dynamic response is analyzed in detail. The main parameters defining the stability state—such as critical dynamic load, critical time, maximum deflection, and buckling mode—are specifically evaluated. The problem is analyzed using two approximation methods: the finite difference method and the finite element method. Numerical calculations were carried out using two approaches: the author’s program following analytical calculations, and the ABAQUS system. The results show the importance of modeling the plate with an appropriate material function describing the radial gradation, which significantly affects the plate’s dynamic stability response and critical parameters.

## 1. Introduction

In the present work, a method for calculating composite annular plates with heterogeneous metal facings is proposed. Numerous analysis results are presented, with primary attention focused on the dynamic responses of plates under loads acting in the plane of the facings. The evaluation of the loss of plate stability, accompanied by a sudden change in the shape of the plate at a specific critical load, shows the sensitivity of the examined structure to its geometrical and material parameters. The application of a heterogeneous structure with smoothly functionally graded facing material enables the construction of new plate structures with a classical three-layer layout whose mechanical properties can meet the required specifications. By evaluating the buckling phenomenon for two material models derived from the radial distribution of the extreme material properties of steel and aluminum, a thorough numerical analysis and discussion of the results have been conducted. The calculation results were obtained for two computational models: an analytical and numerical model based on the finite difference method (FDM) and a numerical model using finite elements. Special attention was given to the computational efficiency of combining analytical and numerical calculations through approximation methods, including orthogonalization and finite differences. The presented calculation techniques and evaluation of buckling behavior complement research in composite plates, creating opportunities for further exploration of structures tailored to specific requirements.

The search for new composite structures that meet technical requirements continues. Plates, including the annular plates examined in this work, are widely used construction elements in various industries, such as mechanical engineering, civil engineering, nuclear, and aerospace applications. Composite three-layered plates with a classic structure, composed of thin metal facings and a thicker foam core, have been extensively analyzed and applied in practice.

The use of a new type of three-layered structure with functional gradation of the facing material may be applied to constructions where increased sensitivity to typical thermo-mechanical loads is expected in the industrial areas mentioned above.

A growing expectation in many industrial sectors is the design of structures dedicated to precise, specific technical solutions. Predicted reactions of elements demonstrating enhanced capabilities of specially composed structures, compared to classical designs, directly fulfill this expectation.

The technical potential of such structures for three-layered annular plates can be realized through the use of heterogeneous elements. Radially variable material properties of the metal facings enable the construction of new layered plates. Recognition of their static and dynamic behaviors, to assess stability loss, is the focus of the research undertaken in this work. It should be emphasized that buckling studies of layered annular plates, including three-layered plates, are presented in numerous scientific works. Thematic groups can be distinguished based on the modeling and load conditions of the examined annular plates. Thermo-mechanical environments are dominant, and most studies focus on plates with transverse material gradation.

The nonlinear bending and post-buckling behavior of a functionally graded material (FGM) annular sector plate is presented in [1]. The plate is composed of two materials: ceramic and metal, with material parameters continuously varying across the plate thickness. The effects of material and geometrical parameters on plate responses have been examined. Two types of boundary conditions have been applied. Numerical calculations were conducted using graded finite elements. The free vibration analysis of the annular sector plate is presented in [2]. The plate is also composed of metal and ceramic. Two theories were used in modeling: first-order shear deformation and Love’s theory. The influence of different geometrical sector ratios and material parameters on the frequencies of the plate sector with various boundary conditions is presented. The dynamic analysis of bidirectional FGMs in a rotating annular plate with variable thickness, including the effects of geometric imperfections and different boundary conditions, is presented in [3]. The FGM plate material parameters vary in both the radial and axial directions. The effect of specific geometrical ratios of the annular plate on low-velocity impact behavior is demonstrated through numerous results. Effects of thermo-mechanical loading, geometrical parameters, and boundary conditions on the large deflection behavior of annular and circular FGM plates are presented in [4]. The material grading through the plate thickness is expressed using a power law. Large deflections are derived from Karman’s equations, which include thermal relations. The frequency analysis of an FGM annular plate under hygrothermal effects, with graded material across the plate thickness, is presented in [5]. Finite element analysis was applied to clamped–clamped and clamped–free boundary conditions for the examined plate model. Free vibration analysis of FGM annular and circular plates is presented in [6]. Material parameters are distributed across the plate thickness according to the power law. The effects of graded material parameters and different geometrical measurements on the natural frequency values are examined. The static temperature analysis of a three-layered annular plate with heterogeneous facings made of material with radially variable parameters is considered in [7]. Material properties are defined by specified exponent functions. The effects of material and geometrical parameters in two plate models, built using FDM and finite element methods (FEM), on the plate response in a thermal environment are shown. The influence of a time-dependent temperature field and the effect of a stationary temperature field on the stability of a three-layered annular plate are presented in [8,9]. Temperature changes, expressed as gradients in the plate’s radial direction, are considered.

Complex loading fields, such as magnetic, electric, and piezo-magnetic, in addition to thermo-mechanical loads, provide additional effects that alter plate responses. Stress and displacement analysis of annular FGP plates subjected to complex loads, including transverse mechanical and magnetic forces, is presented in [10]. Both the elastic modulus and magnetic permeability coefficient vary across the plate thickness according to a power function. Functionally graded piezoelectric annular plates on Winkler foundations are investigated in [11]. The free vibration problem has been solved semi-analytically. The natural frequencies of functionally graded annular plates composed of piezo-magneto-electro-elastic layers are investigated in [12]. The plate is supported on a Pasternak elastic foundation. The plate material is graded through the plate thickness according to a power-law function. The main aim is to investigate the hygrothermal effects. The study examines different parameters, including the thermal environment, boundary conditions, properties of the piezo-magneto-electro-elastic layers, and geometrical ratios.

The behavior of plate elements is also controlled by structural properties, such as porosity. Classical functionally graded annular and circular plates with porosity are examined in [13]. Three porosity models were assumed: uniform, O-shaped, and X-shaped. The analyses focus on the basic natural frequencies. The effects of material and geometrical parameters, as well as porosity distribution, on axisymmetric bending of FGM annular or circular microplates under thermal and mechanical loads are presented in [14]. Nonlinear finite element models with various porosity distributions have been examined.

The effect of porosity in functionally graded materials of sandwich construction elements is presented in [15,16] for beams and pipes, respectively. Material parameters vary across the thickness of the pipe wall or the beam. Complex loads, such as heat transfer, thermo-mechanical loads, or thermo-elastic behavior, are examined.

Geometrical or material heterogeneity creates complex models that require specialized solution techniques. To understand not only the macro-scale impact of structures but also the influence of microstructure on plate responses, tolerance-based modeling techniques are used. These approaches are applied to various problems, including vibrations, stability, thermo-elasticity, heat conduction, and temperature-dependent behavior [17,18,19,20,21,22,23].

The dynamic stability of annular plates subjected to mechanical and thermal loads is presented in [24,25,26,27,28]. The studies by Chen et al. [24] and Wang et al. [25] are exemplary, addressing the dynamic stability analysis of mechanically loaded sandwich annular plates. The dynamic stability of three-layered annular plates subjected to loads acting on the facings is presented in [26,27]. Plate responses to forces increasing over time are analyzed depending on material, geometrical, and loading parameters. Two plate models, built using FDM and FEM, were examined. A buckling analysis of an FGM sandwich square plate with a metal core is presented in [28], examining the effects of geometry, material parameters, and boundary conditions.

The grading of the plate material introduces additional characteristics to the structure, whose response to time-varying loads can vary significantly and depends strongly on the distribution of material properties. The presented review of selected studies on FGM plates demonstrates a wide range of possible applications and research topics. However, there is no existing proposal for a three-layered plate structure with facings made of materials with radially graded properties. The proposed solution distinguishes this type of plate from previously studied plates [8,9,26,27] with homogeneous facings subjected to time-varying, increasing loads. There is a need to develop a new plate model using both the finite element method and a finite difference-based approach. The possibilities of modeling the behavior of a complex system with functionally graded properties, depending on the power-law exponent, are presented in numerous results published in the current study. The effect of mechanical loads and the stationary temperature field, previously analyzed in [7], shows the responses and sensitivity of the new layered plate structure under various conditions. The study demonstrates the potential of using a complex layered system under dynamic conditions. Numerous studies have primarily focused on assessing the impact of material parameters of the outer layers on the plate’s response and provide a foundation for further analyses, such as the influence of the support system or geometric ratios. Experimental studies, which have not yet been conducted, would significantly support the numerical investigations, and their importance is emphasized in the summary of this work.

## 2. Problem Analysis

**Figure 1:**
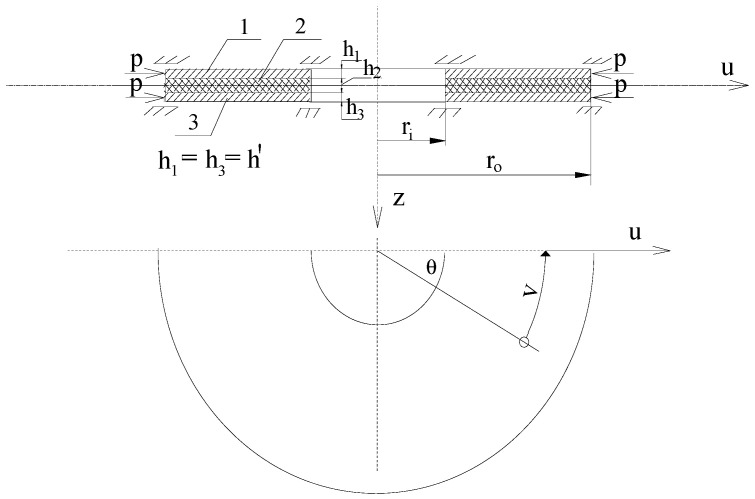
Plate scheme compressed on the outer edge (1, 3—thin facings with thickness *h*′, 2—foam core with thickness *h*_2_) [27].

The object of this analysis is a three-layered annular plate, whose outer layers are made of FGM facings. The plate configuration is shown in Figure 1. It consists of three layers: FGM facings and a homogeneous core, forming a classical sandwich cross-section with thinner facings and a thicker core. The analyzed plate is clamped with sliding supports at both the inner and outer edges.

**Figure 2:**
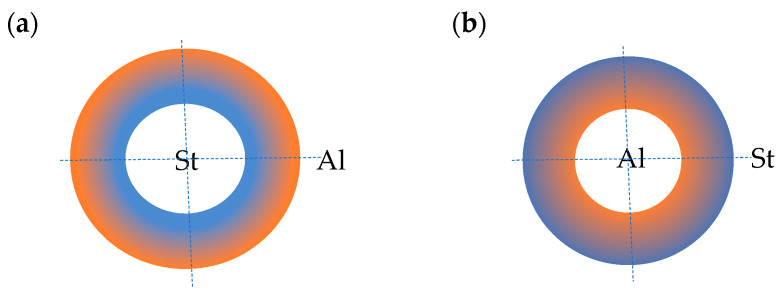
Plate material models: (**a**) St-Al, (**b**) Al-St.

The facings are composed of two metals: steel and aluminum. The material properties vary in the plate’s radial direction according to the functional relation given by Equation (2) for two material models: St-Al, where steel is on the inner edge and aluminum on the outer edge, and Al-St, where aluminum is on the inner edge and steel on the outer edge (see Figure 2).

The analyzed examples include plates with FGM facings and homogeneous facings. The core material is homogeneous, made of polyurethane foam.

The plate is subjected to in-plane forces applied on the inner or outer edge of the facings. In the dynamic analysis, forces increase over time at a specified loading rate. The inner or outer edge is loaded with a uniformly distributed load, linearly increasing in time according to the following formula:

*p* = *st*, (1)

where *p*(*t*) is the compressive stress, *s* is the rate of plate loading, and *t* is time.

The main focus of this study is the buckling behavior of the plate. The plate is initially imperfect and can lose stability in complex modes, with radial and circumferential buckling waves. The primary observed cases are the buckling forms with a half-wave in the radial direction and multiple waves in the circumferential direction, or the axisymmetric form without circumferential waves. Critical dynamic parameters, including the load *p_crdyn_*, time *t_cr_*, deflection *w_cr_*, and mode *m* (defined by the number of circumferential waves), characterize the dynamic stability of the examined FGM plate. The moment of loss of plate stability occurs when the speed of the point of maximum deflection reaches its first peak. This criterion for dynamic stability loss is presented in [29].

## 3. Solution Procedure

The problem is solved using a combination of analytical and numerical calculations. It is based on the orthogonalization method for eliminating the angular variable *θ* (see Figure 1) and the FDM. The classical theory [30] of three-layered plate structures has been adopted. It is based on the assumptions of the broken-line hypothesis and the interaction of stresses between plate layers: normal stresses are carried by the facings, while shear stresses are carried by the core.

Additionally, an FEM model of the plate has been developed using finite elements. The FEM plate structure corresponds to the classical formulation used for the FDM plate model.

### 3.1. Material Model

The radial variation of the FGM facing material is smooth and follows a power-law function:

(2) $$VV={\left(\frac{r_{i}}{r_{i}-r_{o}}+\frac{r}{r_{o}-r_{i}}\right)}^{n},$$

where *VV* is the rate of material variation in the radial direction, *r_i_* and *r_o_* are the plate inner and outer radii, *r* is the radial coordinate, and *n* is the power-law exponent.

The material parameters of the facings, such as Young’s modulus *E_r_*, Kirchhoff’s modulus *G_r_*, Poisson’s ratio *ν_r_*, and mass density *μ_r_* depend on the plate radius *r* and the power-law exponent *n*. They are calculated according to the following equation:

(3) $$W=W1+VV\left(W2-W1\right),$$

where *W*1 and *W*2 are the values of the material parameters (*E_r_*, *G_r_*, *ν_r_*, and *μ_r_*), *W* is the value of the facing material parameters at the plate point determined by the radius *r*. The distribution of material parameters in the facing plane is axisymmetric.

The facings are composed of two materials: steel, with Young’s modulus *E_St_* = 210,000 MPa, and aluminum, with Young’s modulus *E_Al_* = 70,000 MPa, located at the selected edges of the plate. Between the edges, the facings’ material parameters vary according to the selected power-law exponent *n* (see Equation (2)), with *n* = 0.2, 0.5, 1, 2, 5. Figure 16 in Section 4.6 shows the distribution of Young’s modulus along the plate radius for three values of *n* (0.2, 1, 5) for the two plate material models St-Al and Al-St (see Figure 2). The effect of the material model on the stability results is discussed in Section 4.6. The sensitivity of the two plate material models, St-Al and Al-St, is demonstrated by numerous results highlighting the significant impact of the direction of material property gradation relative to the loaded edge of the annular plate. The differences in response are largely explained by the distribution of Young’s modulus in the facing material, determined by different values of the power-law exponent, as shown in Figure 16.

### 3.2. Technique of the Problem Solution Using the FDM

The solution process is based on the method presented in previous works [26,27,31,32]. The solution technique uses both analytical and numerical calculations. The main elements of the solution are as follows:

- The system of dynamic equilibrium equations for the plate layers is established.
- The cross-section of the plate structure is described using the broken-line hypothesis. Geometric relations in the radial and circumferential directions of the plate, expressed by two core angles, are determined.
- Linear physical relations based on Hooke’s law are used to describe the stress state in the plate layers.
- The stress function *Φ* is used to express the resultant membrane forces corresponding to radial and circumferential normal forces and shear forces.
- The transverse radial and circumferential forces in the plate core are expressed.
- The resultant transverse forces for the entire plate are determined.

The analytical calculations lead to the main differential equation for the deflections of the analyzed composite plate in the dynamic problem:

(4) $$\begin{array}{l} N_{1}w_{d,rrrr}+\frac{2N_{1}}{r}w_{d,rrr}-\frac{N_{1}}{r^{2}}w_{d,rr}+\frac{N_{1}}{r^{3}}w_{d,r}+\frac{N_{1}}{r^{4}}w_{d,\theta \theta \theta \theta }+\frac{2\left(N_{1}+N_{2}\right)}{r^{4}}w_{d,\theta \theta }+\\ \frac{2N_{2}}{r^{2}}w_{d,rr\theta \theta }-\frac{2N_{2}}{r^{3}}w_{d,r\theta \theta }-G_{2}\frac{H^{\prime }}{h_{2}}\frac{1}{r}\left(\gamma_{,\theta }+\delta +r\delta_{,r}+H^{\prime }\frac{1}{r}w_{d,\theta \theta }+H^{\prime }w_{d,r}+H^{\prime }rw_{d,rr}\right)\\ =\frac{2h^{\prime }}{r}\left(\frac{2}{r^{2}}\Phi_{,\theta }w_{,r\theta }\right.-\frac{2}{r}\Phi_{,r\theta }w_{,r\theta }+\frac{2}{r^{2}}w_{,\theta }\Phi_{,r\theta }-\frac{2}{r^{3}}\Phi_{,\theta }w_{,\theta }+w_{,r}\Phi_{,rr}+\Phi_{,r}w_{,rr}+\\ \left.\frac{1}{r}\Phi_{,\theta \theta }w_{,rr}+\frac{1}{r}\Phi_{,rr}w_{,\theta \theta }\right)-Mw_{d,\mathrm{tt}}, \end{array}$$

where *w* is the total deflection, *w_d_* is the additional deflection, and *δ*, *γ* are the differences in radial *u*_1_, *u*_3_ and circumferential *v*_1_, *v*_3_ displacements of points on the middle surfaces of the facings, expressed by *δ* = *u*_3_ − *u*_1_ and *γ* = *v*_3_ − *v*_1_, respectively. *N*_1_ = 2*D_r_*, $N_{2}=4D_{{r\theta }_{r}}+\nu_{r}N_{1}$, $D_{r}=\frac{E_{r}h^{3}}{12\left(1-\nu_{r}^{2}\right)}$, $D_{r\theta_{r}}=\frac{G_{r}h^{3}}{12}$ are the rigidities of the plate facings; *h* is the total plate thickness, *H*′ = *h*′ + *h*_2_, where *h*′ is the facings’ thickness and *h*_2_ is the core thickness. *Φ* is the stress function, *M* = 2*h*′*μ*_r_ + *h*_2_, and *μ*_2_ is the core mass density.

Equation (4) was derived after formulating the dynamic equilibrium equations for each plate layer, assuming the deformation of the middle surface of the facings follows the nonlinear von Kármán theory, and describing the transversal geometry of the core deformation based on the linear broken line hypothesis. The equations of Hooke’s law were applied:

(5) $$\sigma_{r_{1(3)}}=\frac{E_{r}}{1-{\nu_{r}}^{2}}(\varepsilon_{r_{1(3)}}+\nu_{r}\varepsilon_{\theta_{1(3)}}),\;\sigma_{\theta_{1(3)}}=\frac{E_{r}}{1-{\nu_{r}}^{2}}\left(\varepsilon_{\theta_{1\left(3\right)}}+\nu_{r}\varepsilon_{r_{1\left(3\right)}}\right),\tau_{r\theta }=G_{r}\gamma_{r\theta },$$

where *σ_r_* is the radial stress, *σ_θ_* is the circumferential stress, and *τ_rθ_* is the shear stress.

Then, the resultant transverse forces in radial and circumferential directions and the resultant membrane forces expressed by the stress function were formulated, assuming the initial conditions that both the additional deflection *w_d_* and the speed of the additional deflection *w*_*d*,*t*_ for time *t* = 0 are equal to zero. Boundary conditions are expressed for the total deflection *w* and the differences in displacements in radial *δ* and circumferential *γ* plate directions:

(6) $${\left.w\right|}_{r=r_{i}(r_{o})}=0,\;{\left.w_{\prime r}\right|}_{r=r_{i}(r_{o})}=0,\;{\left.\delta =\gamma \right|}_{r=r_{i}(r_{o})}=0,\;{\left.\delta_{\prime r}\right|}_{r=r_{i}(r_{o})}=0$$

Loading conditions are defined for the plate edges: inner for radius *r_i_* or outer for radius *r_o_*, respectively:

(7) $${\left.\sigma_{r}\right|}_{r=r_{i}}=-p\left(t\right)d_{1},\;{\left.\sigma_{r}\right|}_{r=r_{o}}=-p\left(t\right)d_{2}$$

The quantities *d*_1_ and *d*_2_ are equal to 0 or 1.

The plate geometry is modeled with preliminary deflections. The shape is determined by combining the axisymmetric component and depends on the number *m* of circumferential waves [33,34]:

(8) $$\zeta_{o}\left(\rho ,\theta \right)=\xi_{1}(\rho )\eta (\rho )+\xi_{2}(\rho )\eta (\rho )\cos(m\theta ),$$

where the dimensionless predeflection is $\zeta_{o}=\frac{w_{o}}{h}$, the dimensionless plate radius is $\rho =\frac{r}{r_{o}}$, *ξ*_1_ and *ξ*_2_ are coefficients that calibrate Equation (8), and *η*(*ρ*) = *ρ*^4^ + *A*_1_*ρ*^2^ + *A*_2_*ρ*^2^*lnρ* + *A*_3_*lnρ* + *A*_4_, with *A_i_* satisfying the conditions for clamped edges.

The shape functions used in the solution of the problem include the additional deflection $\zeta_{1}=\frac{w_{d}}{h}$ [33,34], the functions of the radial and circumferential facings displacements $\bar{\delta }$, $\bar{\gamma }$ [26,27], and the stress function $F=\frac{\Phi }{Eh^{2}}$ [33,34]:

(9) $$\zeta_{1}\left(\rho ,\theta ,t\right)=X_{1}(\rho ,t)\cos(m\theta ),$$

(10) $$\bar{\delta }\left(\rho ,\theta ,t\right)=\bar{\delta }(\rho ,t)\cos(m\theta ),\;\bar{\gamma }\left(\rho ,\theta ,t\right)=\bar{\gamma }(\rho ,t)\sin(m\theta ),$$

(11) $$F(\rho ,\theta ,t)=F_{a}(\rho ,t)+F_{b}(\rho ,t)\cos(m\theta )+F_{c}(\rho ,t)\cos(2m\theta ),$$

where *E* is the established value of the Young’s modulus (*E_St_* for the St-Al plate model or *E_Al_* for the Al-St model), $\bar{\delta }=\frac{\delta }{h},\;\bar{\gamma }=\frac{\gamma }{h}$, and *m* is the number of circumferential waves corresponding to the number of waves shaping the plate imperfection.

The solution technique uses the orthogonalization method to eliminate the angular variable *θ*, along with the FDM, which approximates the derivatives with respect to *ρ* using central differences at discrete points. The main equation of the system of differential equations has the following form [26,27,32]:

(12) $$PU+Q={\ddot{U}}_{K},$$

where the vector ${\ddot{U}}_{K}$ contains elements expressed by the derivative of the additional deflection with respect to time *t*, and the number *K* is given by $K=K7^{2}\cdot \frac{h^{\prime }}{h}\cdot r_{o}h_{2}M$. Here, *K*7 represents the rate of mechanical loading growth calculated as the quotient of *s* (see Equation (1)) to the assumed value of the critical static load *p_cr_*, with $K7=\frac{s}{p_{cr}}$. The vector U contains the additional deflections. The vector Q contains the plate’s material and geometrical parameters, initial and additional deflections, number *m*, and dimensionless radius *ρ*. The matrix P contains the plate’s material and geometrical parameters, number *m*, and parameter *b*, which represent the interval in the FDM.

The Runge–Kutta integration method for the initial state of the plate was used in the numerical solution. Results of the time history of deflection parameters are presented for the dimensionless time t* = *t* · *K*7, which was used in the numerical calculation procedure.

In summary, it should be noted that the presented solution includes several assumptions defining the model of the studied plate, such as the linear material behavior of the plate layers, the neglect of rheological and damping properties of the materials—particularly the foam core—the use of a single type of support system with the plate clamped with sliding supports at the edges, the adopted plate geometry and imperfection shape in the dynamic analysis, and the absence of a functionally variable dynamic loading, including impact loads. The influence of these factors on the final results of plate behavior is possible but does not limit the study of the plate model presented in this work.

### 3.3. Technique of the Problem Solution Using the FEM

The plate model was built using shell and solid elements. Shell elements were used to build the facing mesh, and solid elements were used to build the core mesh. The outer surfaces of the facing mesh elements were connected to the outer surfaces of the core elements using surface contact interaction. Calculations were carried out using the ABAQUS system at the Academic Computer Center CYFRONET-CRACOW via the PLGrid Portal under the awarded grant plgplate1. The Dynamic option of the ABAQUS system was used in the dynamic stability analysis. Static analysis to calculate the critical static load was carried out using the eigenvalue buckling procedure.

## 4. Exemplary Numerical Results

The numerical results are presented for the selected plate models St-Al and Al-St, with various material parameters expressed by the power-law exponent *n*. Results were obtained using two numerical methods: the FDM and the FEM.

### 4.1. Parameters of the FGM Plate Model

Exemplary calculations were carried out for selected geometrical and material plate parameters. Both the facings of the FGM plate and the plate foam core are treated as elastic and isotropic. The accepted data are presented in Table 1.

### 4.2. Accuracy Analysis

**Figure 3:**
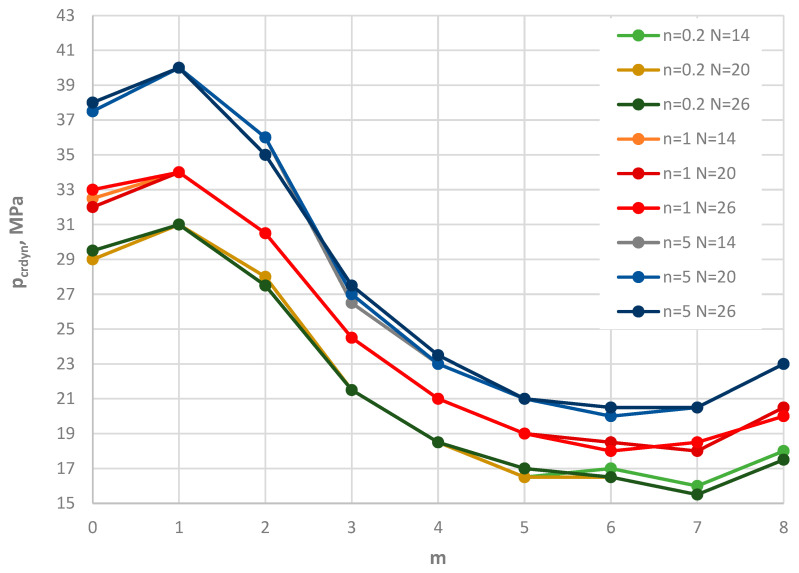
Distribution of the critical dynamic load *p_crdyn_* versus mode number *m* for the St-Al plate model loaded on the outer edge with various values of *n* and number *N* of discrete points.

Figure 3 and Table 2 present the results of the critical dynamic loads, *p_crdyn_*, for various modes *m* for the St-Al plate material model and for three different numbers *N* of discrete points in the FDM: *N* = 14, 20, 26. Three values of the power-law exponent *n* from Equation (2) were considered: *n* = 0.2, 1, 5. The analyzed plates are loaded on the outer edge. The values of *p_crdyn_* show good compatibility for FDM plate models with the asymmetric form of buckling (*m* ≠ 0). Larger differences are observed for the axisymmetric buckling form (*m* = 0). The accuracy between the *p_crdyn_* values for different *N* does not exceed the technical error of approximately 5%. Therefore, *N* = 26 was adopted in the FDM numerical calculations.

Additionally, the results show the following observations:

- The critical dynamic loads *p_crdyn_* are much smaller for the FGM model with power-law exponent *n* = 0.2. A greater proportion of steel increases the critical load.
- The minimal values of *p_crdyn_* occur for plates with several circumferential buckling waves, *m* = 6 or 7.
- There are significant differences between the *p*_crdyn_ values for plates without circumferential buckling waves (*m* = 0) and those with *m* corresponding to the minimal value of *p_crdyn_*. This confirms the importance of the complex solution procedure, which includes asymmetric plate cases.

### 4.3. Plate Loaded on the Outer Edge

**Figure 4:**
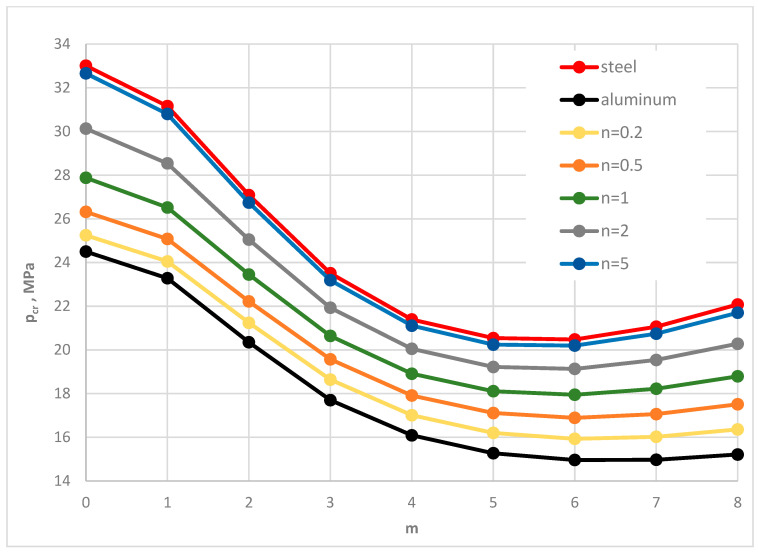
Distribution of the critical static load *p_cr_* versus mode number *m* for St-Al, steel, and aluminum plate models.

Figure 4 shows the distribution of the critical static load *p_cr_* versus the plate mode *m* for different facing materials: homogeneous steel, homogeneous aluminum, and FGM with different values of the power-law exponent *n*. Numerical results were obtained using the FDM for St-Al facing models. The plate is loaded on the outer edge. The distribution of results shows a regular pattern. As the steel content in the FGM facings increases, the critical static load *p_cr_* also increases. The results for the FGM with *n* = 5 are similar to those for homogeneous steel facings. This FGM-foam-FGM structure is lighter than a steel–foam–steel structure, without significantly reducing the stability parameters. Static analysis complements the understanding of the dynamic responses of the examined composite plate.

**Figure 5:**
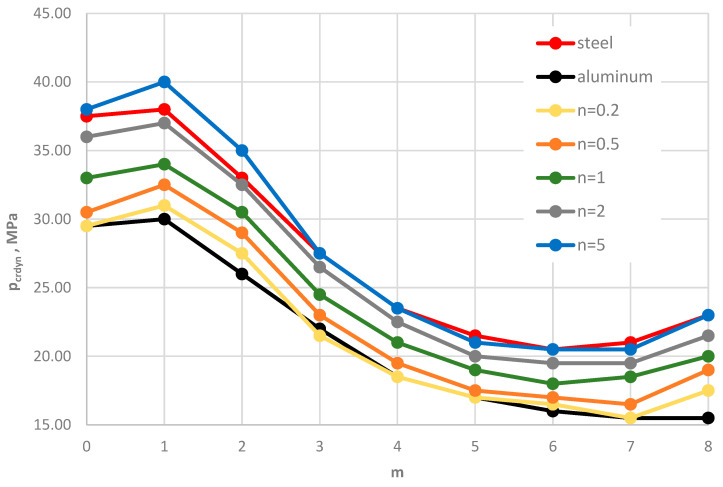
Distribution of the critical dynamic load *p_crdyn_* versus mode number *m* for St-Al, steel, and aluminum plate models.

Similar observations for dynamically loaded plates are shown in Figure 5. The critical dynamic loads, *p_crdyn_*, for corresponding modes *m* are presented for St-Al facing models with different material distributions expressed by the power-law exponent *n*. For plates with steel facings (St-Al material, *n* = 5) and plates with aluminum facings (St-Al material, *n* = 0.2), the critical dynamic load values are particularly close for plates with larger modes m. For these examples, the sensitivity of the dynamic stability appears to be smaller. There are no significant differences between results for plates composed of homogeneous layers and the examined heterogeneous ones, especially for plates with minimal values of *p_crdyn_*.

The comparison between the critical dynamic loads, *p_crdyn_*, and static loads, *p_cr_*, is presented in Table 3. Results are shown for homogeneous plate structures and FGM facings with three power-law exponents, *n* = 0.2, 1, 5. Greater differences are observed for the axisymmetric buckling form (*m* = 0) and for plate structures with aluminum facings or heterogeneous facings containing a lower proportion of steel material (*n* = 0.2). Close values are observed for asymmetric modes with multiple circumferential waves, particularly for the mode corresponding to minimal values of critical loads (*m* = 6). Slightly higher values are observed for dynamically loaded plates. This allows the conclusion that static analysis is sufficient for evaluating the stability of FGM-foam-FGM plates loaded on the outer edge.

**Figure 6:**
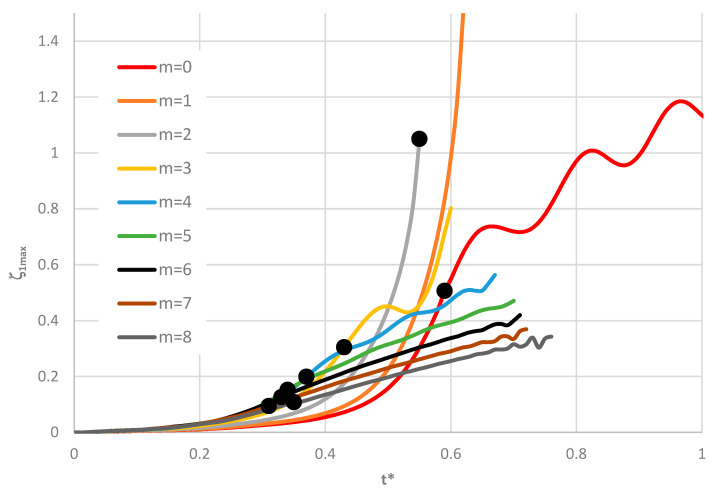
Time history of dimensionless deflections *ζ*_1*max*_ depending on St-Al plate mode *m* for *n* = 0.2.

**Figure 7:**
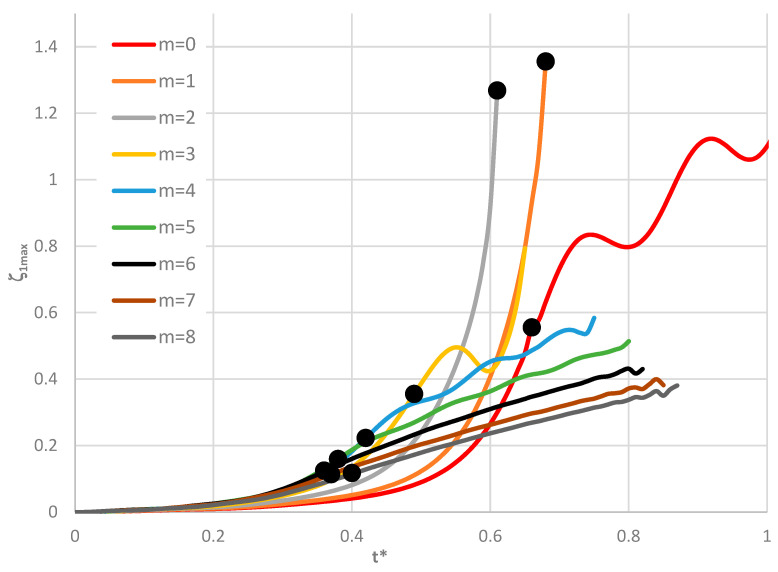
Time history of dimensionless deflections *ζ*_1*max*_ depending on St-Al plate mode *m* for *n* = 1.

**Figure 8:**
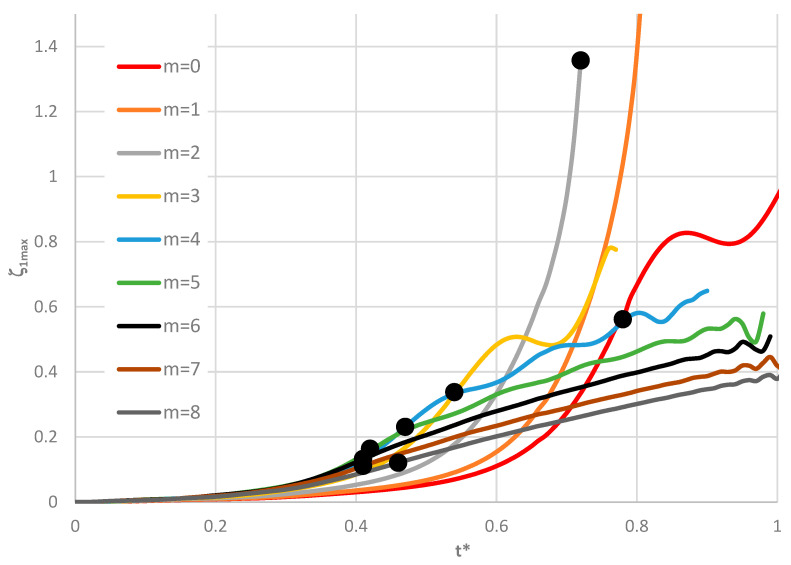
Time history of dimensionless deflections *ζ*_1*max*_ depending on St-Al plate mode *m* for *n* = 5.

Time histories of plate deflections for various modes are shown in Figure 6, Figure 7 and Figure 8. The curves were obtained for three values of the power-law exponent, *n* = 0.2, 1, 5. The highlighted dots indicate the moment of the plate’s loss of stability according to the adopted criterion. The results show two groups of curves: one for *m* < 4, corresponding to fewer than four circumferential waves, and another for *m* > 3. Differences are observed in the character of the curves, the distribution of points representing the moment of loss of plate stability, and in the overcritical area, where vibrations can be initiated.

In summary, a noticeable increase in the values of the critical dynamic load, *p_crdyn_*, is observed with an increase in the power-law exponent *n*. The minimum values consistently occur for plates exhibiting significant circumferential waves. The differences in *p_crdyn_* between plates with power-law exponent values of *n* = 0.2, 1, and 5 are approximately 3 MPa and 2 MPa, respectively (see Table 3).

### 4.4. Plate Loaded on the Inner Edge

**Figure 9:**
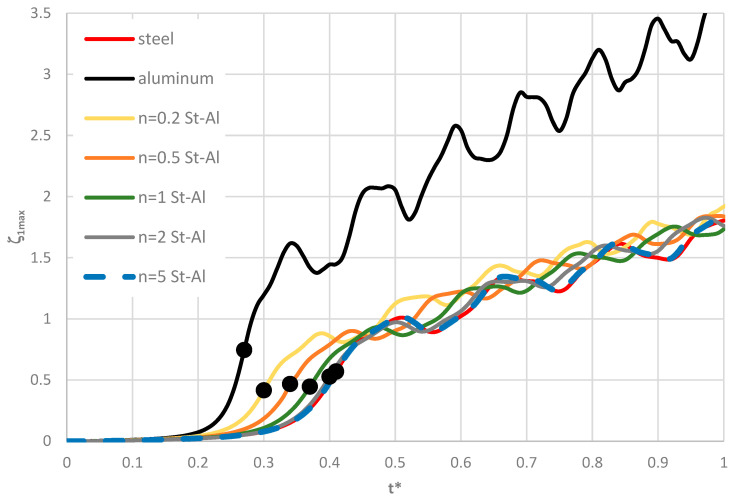
Time history of dimensionless deflections *ζ*_1*max*_ depending on steel, aluminum, and St-Al plate models for various power-law exponents *n* and plate mode *m* = 0.

**Figure 10:**
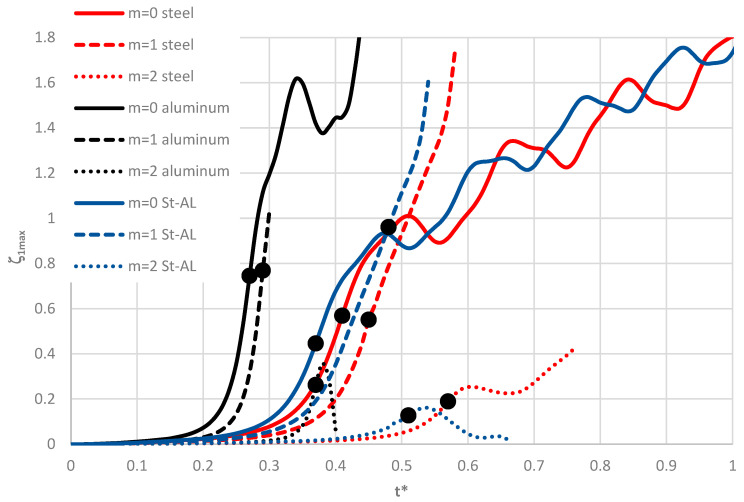
Time history of dimensionless deflections *ζ*_1*max*_ depending on steel, aluminum, and St-Al plate models for power-law exponent *n* = 1 and modes *m* = 0, 1, 2.

Exemplary results for the St-Al plate model loaded on the inner edge are shown in Figure 9 and Figure 10. The time histories of deflections for axisymmetric (*m* = 0) homogeneous plates and plates with FGM facings, presented in Figure 9, indicate similar dynamic responses for plates with steel–aluminum or steel facings. Increasing the steel content in the facings prolongs the time to the loss of plate stability and increases the dynamic critical load, *p_crdyn_*. The homogeneous plate with aluminum facings has less favorable stability parameters: the critical load is smaller, the critical deflection is higher, and overcritical vibrations with greater amplitude occur compared to the St-Al plate model.

Curves in Figure 10 confirm that the minimal values of *p_crdyn_* for plates loaded on the inner edge correspond to the axisymmetric buckling form (*m* = 0). Results were obtained for plate modes *m* = 0, 1, and 2. The analysis considers both homogeneous plates and plates with St-Al facings with *n* = 1. The detailed values of *p_crdyn_* for axisymmetric (*m* = 0) plates are presented in Table 5 in Section 4.5 of this paper The difference between St-Al plates with material distributions corresponding to *n* = 0.2 and *n* = 5 is significant. Increasing the steel content in the facings is more advantageous for the mechanical stability of the plates.

In summary, based on the presented calculation results, a significant difference is observed between plates with aluminum facings and heterogeneous plates defined by a power-law exponent of *n* = 0.2. The difference in behavior between these two plate types is particularly noticeable in the overcritical region, where vibrations are initiated (see Figure 9).

### 4.5. Results for Both FDM and FEM Plate Models

**Figure 11:**
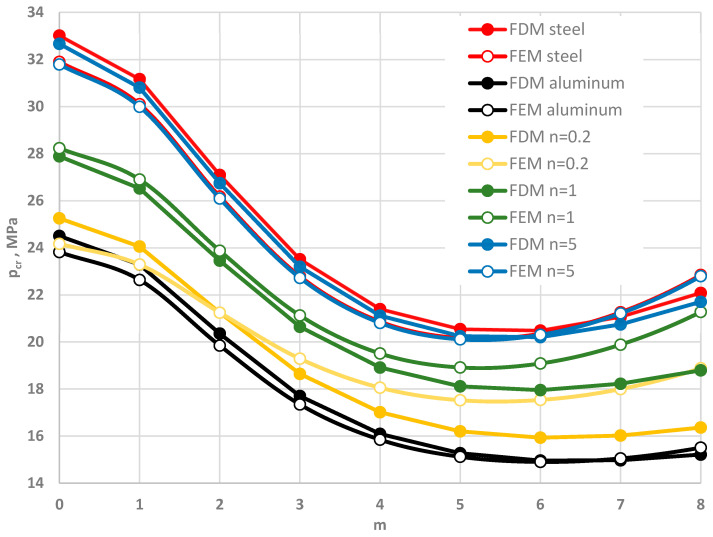
Distribution of the critical static load *p_cr_* versus mode number *m* for FDM and FEM plate models with homogeneous and St-Al facings with *n* = 0.2, 1, 5.

The comparison between the two plate models, FDM and FEM, is presented in Figure 11 and Table 4 and Table 5. Figure 11 shows the critical static loads, *p_cr_*, for FDM and FEM plate models. Good agreement of *p_cr_* values is observed for homogeneous plates, St-Al facings with *n* = 5, and heterogeneous plates with mode numbers *m* < 5. For higher modes, differences in *p_cr_* increase for plates with facing materials characterized by power-law exponents *n* = 0.2 and *n* = 1. In these cases, lower *p_cr_* values are observed for the FDM plate model.

The values of *p_crdyn_* for asymmetric plate modes are closer. Detailed values are presented in Table 4. Greater differences are observed for axisymmetric plates (*m* = 0). Table 5 shows the values of *p_crdyn_* for plates loaded on the inner edge. Good agreement is observed for plate cases with increased steel content in the facings and for homogeneous plates. Greater differences occur between FDM and FEM plate models with St-Al facings at *n* = 0.2.

**Figure 12:**
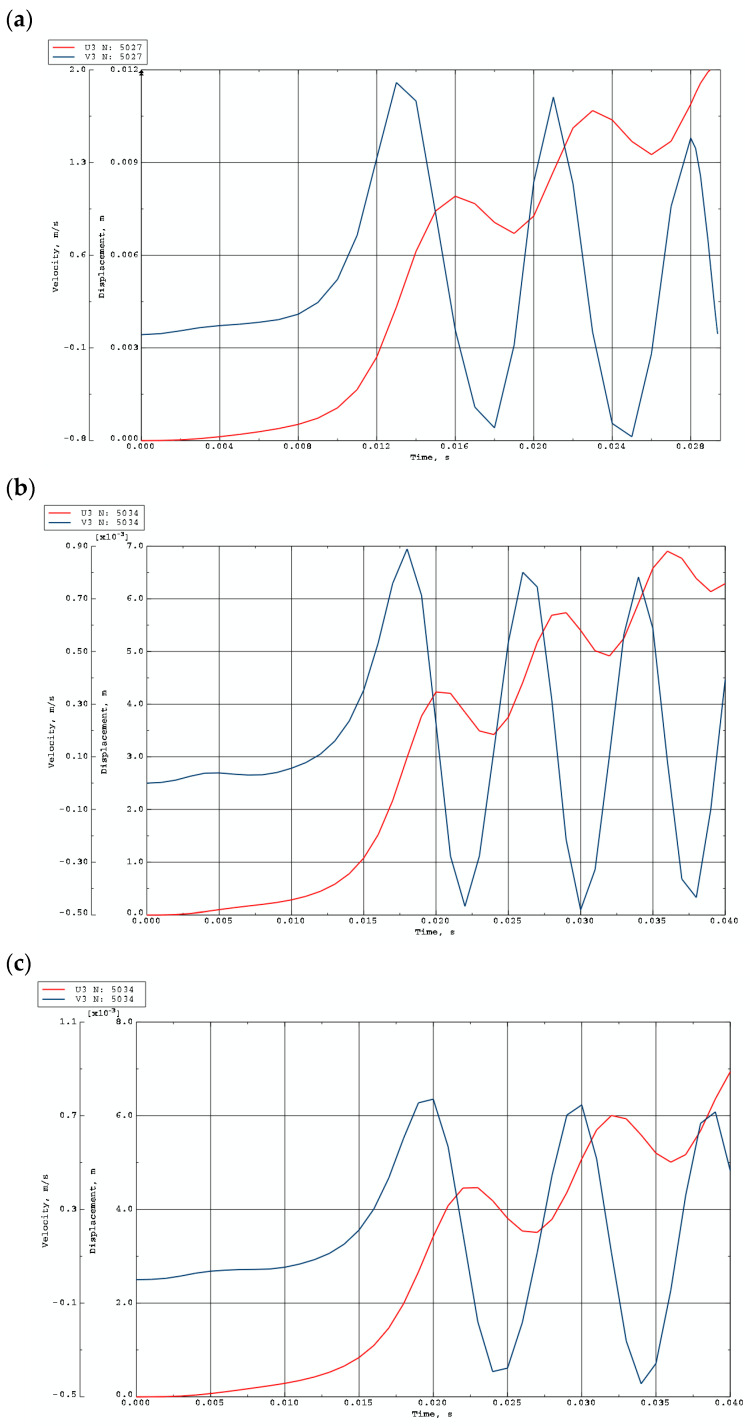
Time histories of deflections and velocity of deflections for plates with *m* = 0 loaded on the inner edge: (**a**) aluminum plate model, (**b**) St-Al plate model with *n* = 0.2, (**c**) steel plate model.

**Figure 13:**
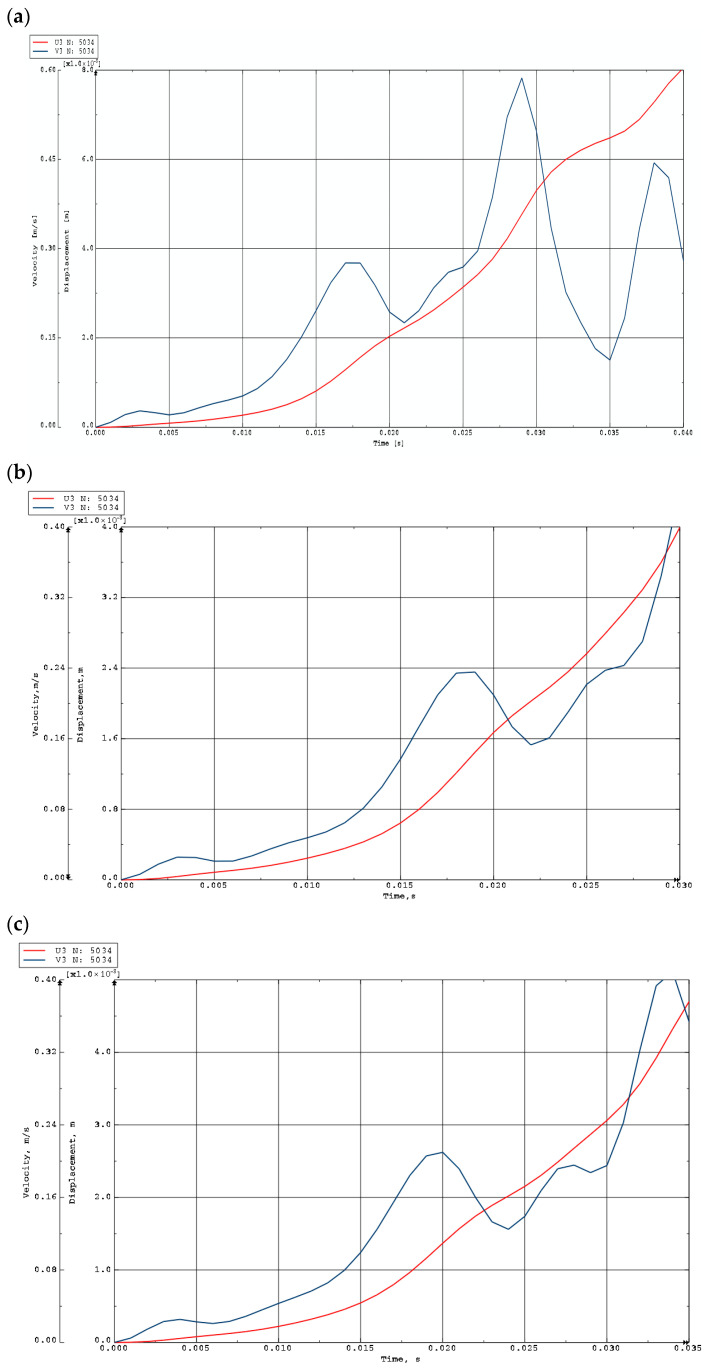

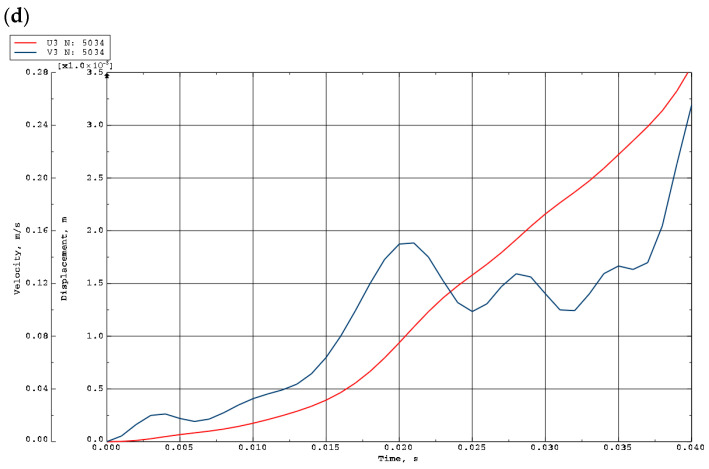
Time histories of deflections and velocity of deflections for plates with *m* = 6 loaded on the outer edge: (**a**) St-Al plate model with *n* = 0.2, (**b**) St-Al plate model with *n* = 1, (**c**) St-Al plate model with *n* = 5, (**d**) steel plate model.

Exemplary time histories of deflections (red lines) and velocities of deflections (blue lines) for FEM plate models are shown in Figure 12 and Figure 13. Figure 12 presents the curves for homogeneous plates and St-Al plates with *n* = 0.2, loaded on the inner edge. The curves confirm the tendency toward the initiation of overcritical vibrations. The first maximum of the velocity time history indicates the moment of loss of dynamic stability. Figure 13 shows the response of FEM plate models with different material parameters: St-Al plate facings with *n* = 0.2, 1, 5, and a plate model with steel facings. Results are presented for mode *m* = 6. No vibrations are observed in the overcritical area.

**Figure 14:**
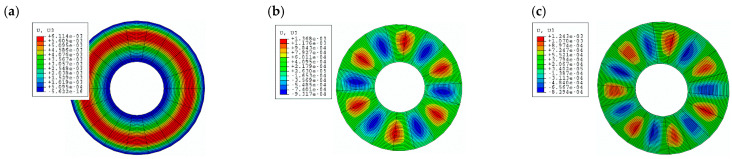
Exemplary forms of plate buckling loaded on the outer edge: (**a**) axisymmetric *m* = 0 St-Al model with *n* = 5, (**b**) asymmetric *m* = 6 St-Al model with *n* = 5, (**c**) asymmetric *m* = 7 St-Al model with *n* = 1.

Exemplary forms of buckling—axisymmetric *m* = 0 and asymmetric with *m* = 6 and *m* = 7 circumferential waves for plates loaded on the outer edge—are shown in Figure 14.

An additional comparison of the critical static loads, *p_cr_*, determined by the FDM and FEMs for three-layered plates with homogeneous steel or aluminum facings is presented in Table 6. The calculated *p_cr_* values were compared with those determined for the FEM model presented in [35]. The results show good agreement between the critical static loads of the analyzed FDM and FEM numerical models and the FEM model from [35] for axisymmetric (*m* = 0) plates loaded on the outer edge.

### 4.6. Analysis of Results for Two St-Al and Al-St FGM Plate Models

**Figure 15:**
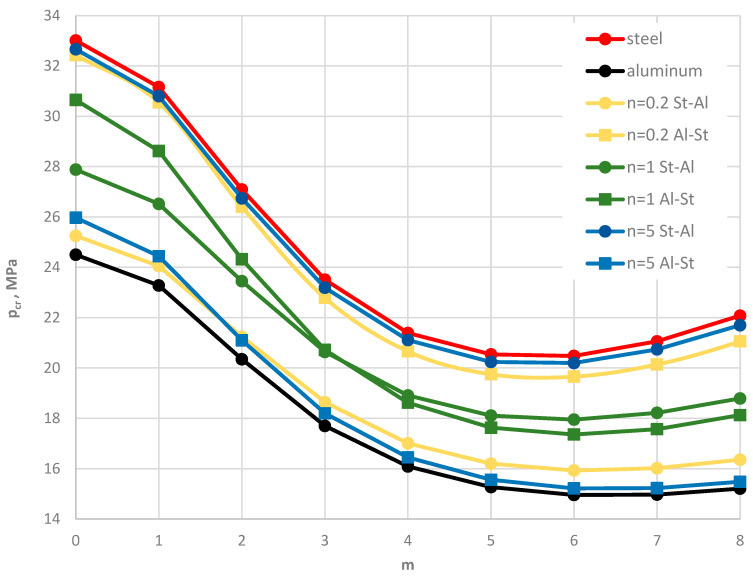
Distribution of the critical static load *p_cr_* versus mode number *m* for St-Al and Al-St FDM plate models, both homogeneous and with FGM facings, for power-law exponents *n* = 0.2, 1, 5.

**Figure 16:**
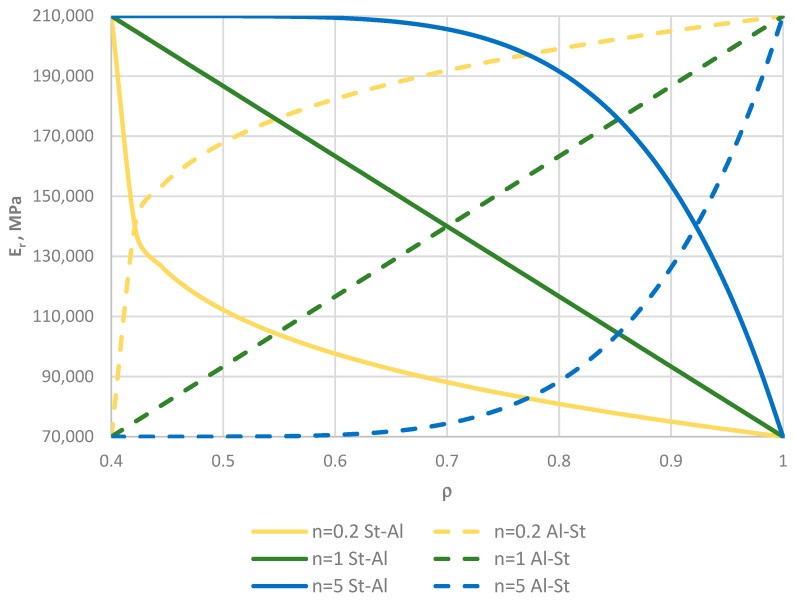
Distribution of Young’s modulus *E_r_* of St-Al and Al-St FGM facings versus plate radius *ρ*.

**Figure 17:**
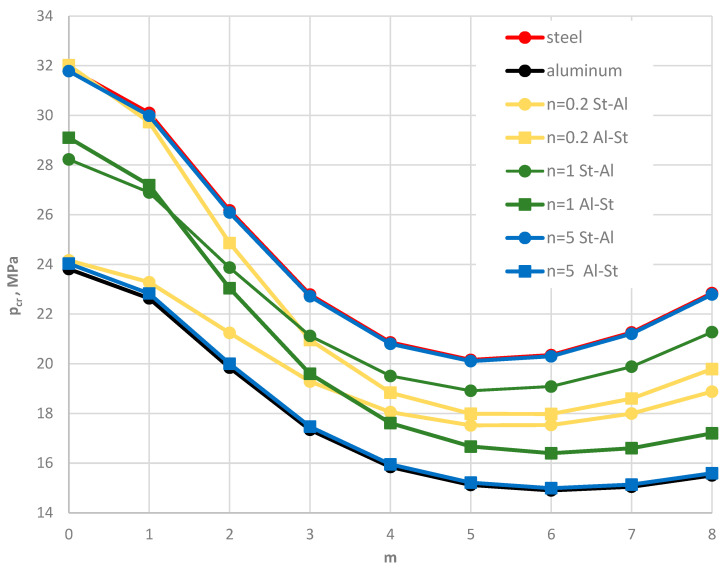
Distribution of the critical static load *p_cr_* versus mode number *m* for homogeneous, St-Al, and Al-St FEM plate models, with power-law exponents *n* = 0.2, 1, 5.

**Figure 18:**
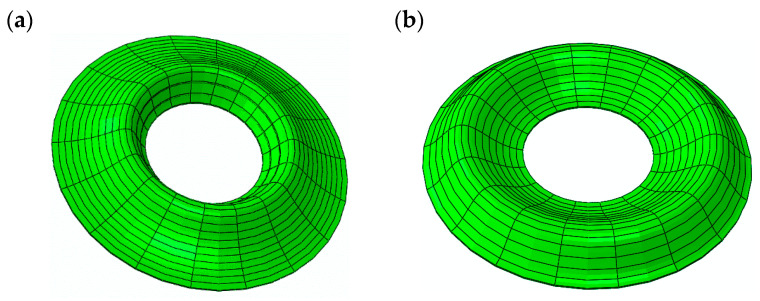
Axisymmetric *m* = 0 buckling modes for the FEM plate model loaded on (**a**) inner edge, (**b**) outer edge.

**Figure 19:**
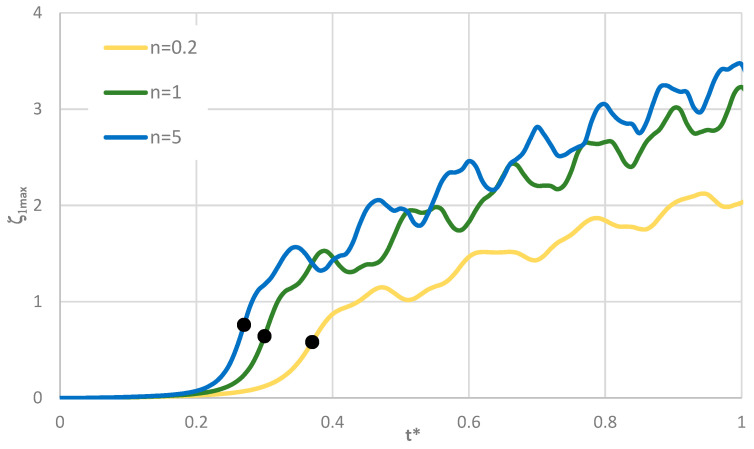
Time history of dimensionless deflections *ζ*_1*max*_ versus power-law exponent *n* for FDM Al-St plate models loaded on the inner edge.

Results for the two plate models, St-Al and Al-St (see Figure 2), are presented in Figure 15, Figure 16, Figure 17, Figure 18 and Figure 19 and Table 7. The computational analyses for the two plate material models focus on demonstrating the effect of FGM arrangement in the plate facings on the critical static loads, *p_cr_*, and dynamic loads, *p_crdyn_*. Differences in behavior are observed. Figure 15 shows the critical static load, *p_cr_*, for St-Al and Al-St FDM plate models loaded on the outer edge. The plate models exhibit opposite responses. The *p_cr_* values for the Al-St plate with *n* = 5, where aluminum is located near the inner plate edge, are the smallest, whereas for *n* = 0.2, the critical loads are close to those for a homogeneous steel plate. The FGM distribution defined by Equations (2) and (3) with different values of *n* for the Al-St plate, shown in Figure 16, explains these changes. The softer aluminum material is concentrated near the inner edge for *n* = 5, resulting in generally smaller material parameters compared to *n* = 0.2. Results obtained using FEM do not show such clear differences for the FGM plate model with *n* = 0.2 (see Figure 17). For asymmetric modes, the *p_cr_* values for both St-Al and Al-St plate models converge. The opposite response trend for the two models is still observed. It can be concluded that, from a stability point of view, the lighter Al-St material distributed in the plate facings is more advantageous, providing higher critical static loads, *p_cr_*, for plates loaded on the outer edge.

**Figure 20:**
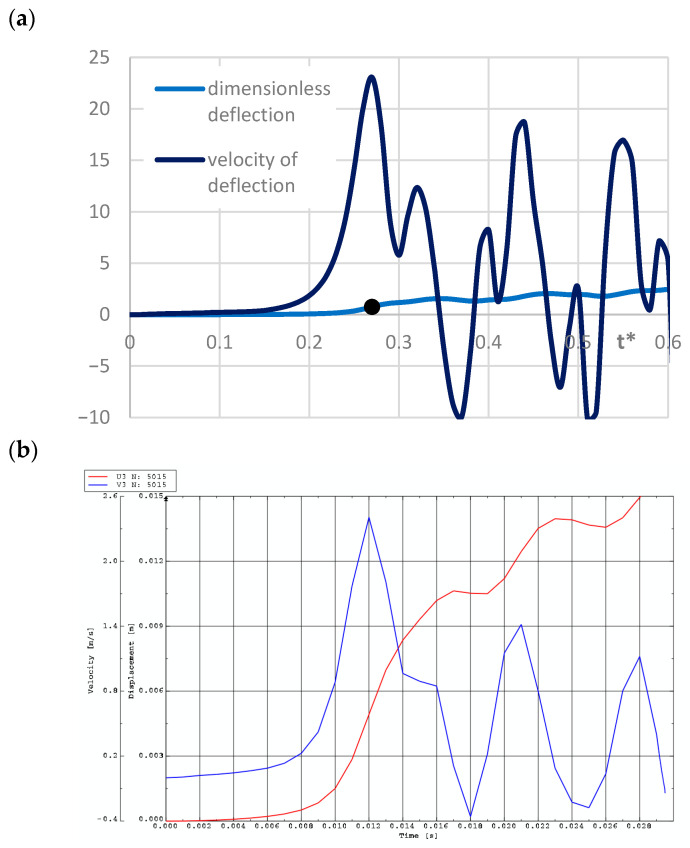
Dynamic results for the Al-St plate (*m* = 0) with *n* = 5 loaded on the inner edge: (**a**) dimensionless deflections and velocities for the FDM plate model; (**b**) deflections and velocities for the FEM plate model.

The forms of axisymmetric buckling of plates loaded on the inner and outer edges are shown in Figure 18. Differences in the critical deformations of the plate structure are visible between plates loaded on the inner and outer edges. This axisymmetric form is particularly important for plates loaded on the inner edge. This axisymmetric *m* = 0 mode corresponds to the plate model with the smallest critical load. The time history of dimensionless deflections presented in Figure 19 for the FDM Al-St plate model loaded dynamically on the inner edge confirms the results obtained for the Al-St plate model loaded statically. The shortest time to loss of plate stability, identifiable by the black dots, and the smallest value of the critical dynamic load *p_crdyn_* occur for the plate model with *n* = 5. Larger stability parameters are observed for the plate with *n* = 0.2. Detailed values for the two material plate models, St-Al and Al-St, for both FDM and FEM computational models, are presented in Table 7. Plates are loaded statically and dynamically on the inner edge. For the FGM plate with *n* = 5, whose critical load values are close to those of a homogeneous steel or aluminum composite plate, the results depend on the material model (St-Al or Al-St). Detailed results show good agreement between the two computational models, FDM and FEM. Critical loads of the St-Al plate model are close to those of a homogeneous steel plate, while values obtained for the Al-St plate model are close to the critical loads for plates with aluminum facings. Considering the detailed values of *p_cr_* and *p_crdyn_* calculated for the plate model with *n* = 5, the St-Al material plate model, for this specific material distribution, is more advantageous from a stability point of view for both inner and outer-edge loads. The values of *p_cr_* and *p_crdyn_* are close to those of a composite plate with steel facings. Exemplary time histories of deflections and deflection velocities for the two computational models, FDM and FEM, are presented in Figure 20. The Al-St plate model with *n* = 5 is loaded on the inner edge. For the FDM plate model, results are presented in dimensionless form (see Figure 20a). For example, dimensionless time t* is related to real time *t* (Figure 20b) by the formula t* = *tK*7, where parameter *K*7 equals 20. The character of the presented curves for both FDM and FEM plate models is comparable. Characteristic overcritical vibrations, initiated by the increasing applied load, are observed.

## 5. Conclusions

This paper presents the dynamic responses of composite annular plates with facings made of FGM. Alternating material property distributions create two types of plate models with facings of steel–aluminum or aluminum–steel. In these models, steel or aluminum is located at the inner edge, while aluminum or steel is positioned at the outer edge, respectively. Radial changes in the material parameters are determined by a power function, in which the power-law exponent *n* takes the values *n* = 0.2, 0.5, 1, 2, 5. Numerical calculations were performed using two methods: FDM and FEM.

The numerical results are presented with particular attention to the evaluation of the plate’s critical-state parameters. The main observations concern the critical dynamic loads, which are complemented by the evaluation of changes in the values determined for the static state of the plate, namely the critical static loads. The discussion of the calculation results highlights the importance of the radial distribution of the facing material in relation to the type of plate-edge loading: inner or outer. To identify composite plate structures with greater resistance to buckling, attention is focused on cases exhibiting higher critical parameter values. Specifically, maximum values of *p_crdyn_* and *p_cr_* are observed when steel predominates in the facings. The most suitable model is the St-Al plate with a power-law exponent of *n* = 5 under outer-edge loading, or a plate with *n* = 0.2 under inner-edge loading. This opposite trend arises because the stiffer and softer materials are located differently relative to the loaded edge. A key benefit of using FGMs with radially variable parameters is a significant reduction in plate weight, with only a slight reduction in stability-related properties.

The computational models, built according to the assumptions and limitations of the applied approximation methods, were tested. The static and dynamic responses of the FDM and FEM plate models show good agreement. Observed differences in critical load values mainly concern the plate with *n* = 0.2, whose facings are more flexible due to the increased contribution of aluminum parameters. The proposed analytical and numerical FDM solution procedure demonstrates techniques for modeling complex plate structures and enables rapid, effective calculations for variable plate parameters. From a practical point of view, the effectiveness of the proposed procedure is high.

It is possible to extend the analyses to cases of plates whose facing material functions have various forms. The presented numerical results should be complemented by future experimental studies.

The dynamic response of plates with varying property gradation, represented by the St-Al and Al-St models, is particularly interesting. The opposite responses of the St-Al and Al-St models highlight the strong influence of material ordering on both static and dynamic stability. This is especially evident when examining the noticeable differences between FDM and FEM results for plates with significant circumferential waves.

Additionally, the consideration of a heterogeneous material for the plate core could significantly enrich the evaluation of the examined composite plates. In practice, selecting the FGM facing arrangement and power-law exponent according to edge-loading conditions can optimize stability while minimizing plate weight, providing a clear guideline for lightweight composite plate design. Although numerical results are consistent across methods, experimental validation is recommended to confirm these findings under real loading conditions. This topic will be addressed in future work.

## Figures and Tables

**Table 1:** Geometrical and material plate parameters.

Description	Parameter	Value
Geometrical parameters		
plate inner radius	*r_i_*	0.2 m
plate outer radius	*r_o_*	0.5 m
facing thickness	*h*′	1 mm
core thickness	*h*_2_	5 mm
Material parameters		
Young’s modulus of steel	*E_st_*	210 GPa
Poisson’s ratio of steel	*ν_st_*	0.3
mass density of steel	*µ_st_*	7850 kg/m^3^
Young’s modulus of aluminum	*E_al_*	70 GPa
Poisson’s ratio of aluminum	*ν_al_*	0.33
mass density of aluminum	*µ_al_*	2700 kg/m^3^
Kirchhoff’s modulus of polyurethane foam of core material	*G_c_*	5 MPa
mass density of polyurethan foam of core material	*µ_c_*	64 kg/m^3^
power-law exponent of Equation (2)	*n*	0.2, 0.5, 1, 2, 5
Load parameters		
rate of dynamic loading growth	*s*	1000 MPa/s for plate loaded on the outer edge
		5000 MPa/s for plate loaded on the inner edge

**Table 2:** Detailed values of the critical dynamic loads *p_crdyn_* versus mode number *m* for the St-Al plate model loaded on the outer edge for different values of *n* and number *N* of discrete points.

*m*	*p_crdyn_*, MPa					
	St-Al Model *n* = 1			St-Al Model *n* = 5		
	*N* = 14	*N* = 20	*N* = 26	*N* = 14	*N* = 20	*N* = 26
0	32.5	32	33	37.5	37.5	38
1	34	34	34	40	40	40
2	30.5	30.5	30.5	36	36	35
3	24.5	24.5	24.5	26.5	27	27,5
4	21	21	21	23	23	23.5
5	19	19	19	21	21	21
6	18.5	18.5	18	20	20	20.5
7	18	18	18.5	20.5	20.5	20.5
8	20.5	20.5	20	23	23	23

**Table 3:** Critical dynamic *p_crdyn_* and static *p_cr_* loads for the FDM plate model.

*m*	FDM Plate Model									
	Steel		Aluminum		St-Al *n* = 0.2		St-Al *n* = 1		St-Al *n* = 5	
	*p_crdyn_*, MPa	*p_cr_*, MPa	*p_crdyn_*, MPa	*p_cr_*, MPa	*p_crdyn_*, MPa	*p_cr_*, MPa	*p_crdyn_*, MPa	*p_cr_*, MPa	*p_crdyn_*, MPa	*p_cr_*, MPa
0	37.5	33.01	29.5	24.5	29.5	25.25	33	27.88	38	32.66
4	23.5	21.39	18.5	16.09	18.5	17.01	21	18.91	23.5	21.11
5	21.5	20.54	17	15.27	17	16.20	19	18.11	21	20.24
6	20.5	20.48	16	14.96	16.5	15.93	18	17.95	20.5	20.20
7	21	21.06	15.5	14.97	15.5	16.02	18.5	18.22	20.5	20.74

**Table 4:** Results for FDM and FEM plate models loaded on the outer edge.

*m*	*p_crdyn_*, MPa									
	Plate Model									
	Steel		Aluminum		St-Al *n* = 0.2		St-Al *n* = 1		St-Al *n* = 5	
	FDM	FEM	FDM	FEM	FDM	FEM	FDM	FEM	FDM	FEM
0	37.5	37	29.5	21	29.5	27	33	29	38	33
4	23.5	24	18.5	19	18.5	20	21	21	23.5	22
5	21.5	22	17	17	17	18	19	19	21	20
6	20.5	21	16	16	16.5	17	18	19	20.5	20
7	21	22	15.5	16	15.5	18	18.5	19	20.5	20

**Table 5:** Results for FDM and FEM plate models loaded on the inner edge.

*m*	*p_crdyn_*, MPa									
	Plate Model									
	Steel		Aluminum		St-Al *n* = 0.2		St-Al *n* = 1		St-Al *n* = 5	
	FDM	FEM	FDM	FEM	FDM	FEM	FDM	FEM	FDM	FEM
0	102.5	100	67.5	65	77.5	90	92.5	100	100	100

**Table 6:** Comparison of critical static load values *p_cr_* between FDM and FEM plate models and the FEM model in [35] for axisymmetric plates loaded on the outer edge.

*p_cr_*, MPa		
Three-layered plate with homogeneous steel facings		
FDM model	FEM model	FEM model in [35]
33.01	31.89	32.81
Three-layered plate with homogeneous aluminum facings		
FDM model	FEM model	FEM model in [35]
24.5	23.82	24.39

**Table 7:** Critical static *p_cr_* and dynamic *p_crdyn_* loads for St-Al and Al-St FDM and FEM plate models loaded on the inner edge.

*m* = 0	Homogeneous Plate Model				FGM Plate Model with *n* = 5			
	Steel		Aluminum		St-Al		Al-St	
	FDM	FEM	FDM	FEM	FDM	FEM	FDM	FEM
*p_cr_*, MPa	76.57	71.78	49.23	46.75	76.56	71.83	49.36	46.67
*p_crdyn_*, MPa	102.5	100	67.5	65	100	95	67.5	60

## Data Availability

The original contributions presented in this study are included in the article. Further inquiries can be directed to the corresponding author.

## Abbreviations

Abbreviations	
FGM	functionally graded material
FDM	finite difference method
FEM	finite element method
St-Al	plate material model with steel on the inner edge and aluminum on the outer edge
Al-St	plate material model with aluminum on the inner edge and steel on the outer edge
Main parameters	
*r_i_*	plate inner radius
*r_o_*	plate outer radius
*r*	plate radius
*ρ_i_*	dimensionless plate inner radius
*h*′	facing thickness
*h*_2_	core thickness
*E_St_*	Young’s modulus of steel
*E_Al_*	Young’s modulus of aluminum
*μ_St_*	mass density of steel
*μ_Al_*	mass density of aluminum
*n*	FGM-facing material power-law exponent (see Equation (2))
*G*_2_	Kirchhoff’s modulus of polyurethane foam as core material
*N*	number of discretization points
*p_crdyn_*	critical dynamic load
*t_cr_*	critical time
*t**	dimensionless time
*ζ*_1*max*_	dimensionless additional deflection
*p_cr_*	critical static load
*m*	plate buckling mode

## References

[1] Asemi K., Salehi M., Akhlaghi M. (2014). Post-buckling analysis of FGM annular sector plates based on three dimensional elasticity graded finite elements. Int. J. Non-Linear Mech..

[2] Civalek Ö., Baltacıoglu A.K. (2019). Free vibration analysis of laminated and FGM composite annular sector plates. Compos. Part B.

[3] Fan Y.-H., She G.-L. (2026). Low-velocity impact response of rotating 2DFGM annular plates with variable thickness. Commun. Nonlinear Sci. Numer. Simul..

[4] Golmakani M.E., Kadkhodayan M. (2011). Large deflection analysis of circular and annular FGM plates under thermo-mechanical loadings with temperature-dependent properties. Compos. Part B.

[5] Sharma P., Singh R. (2019). Investigation on modal behaviour of FGM annular plate under hygrothermal effect. IOP Conf. Ser. Mater. Sci. Eng..

[6] Khare S., Vishwakarma R., Vasara D., Kumar R. (2022). Prediction of natural frequencies of functionally graded circular and annular plate via differential quadrature method (DQM). ASPS Conf. Proc..

[7] Pawlus D. (2024). Three-Layered Annular Plate Made of Functionally Graded Material Under a Static Temperature Field. Materials.

[8] Pawlus D. (2020). Dynamic response of three-layered annular plates in time-dependent temperature field. Int. J. Struct. Stab. Dyn..

[9] Pawlus D. (2019). Stability of three-layered annular plate in stationary temperature field. Thin-Walled Struct..

[10] Shishesaz M., Zakipour A., Jafarzadeh A. (2016). Magneto-Elastic Analysis of an Annular FGM Plate Based on Classical Plate Theory Using GDQ Method. Lat. Am. J. Solids Struct..

[11] Yas M.H., Jodaei A., Irandoust S., Nasiri Aghdam M. (2012). Three-dimensional free vibration analysis of functionally graded piezoelectric annular plates on elastic foundations. Meccanica.

[12] Kiarasi F., Babaei M., Asemi K., Dimitri R., Tornabene F. (2022). Free Vibration Analysis of Thick Annular Functionally Graded Plate Integrated with Piezo-Magneto-Electro-Elastic Layers in a Hygrothermal Environment. Appl. Sci..

[13] Vasara D., Khare S., Sharma H.K., Kumar R. (2022). Free vibration analysis of functionally graded porous circular and annular plates using differential quadrature method. Forces Mech..

[14] Kim J., Nava E., Rakici S. (2023). Nonlinear Finite Element Model for Bending Analysis of Functionally-Graded Porous Circular/Annular Micro-Plates under Thermomechanical Loads Using Quasi-3D Reddy Third-Order Plate Theory. Materials.

[15] Zhang Z., Sun Y., Cao X., Xu J., Yao L. (2024). A slice model for thermoelastic analysis of porous functionally graded material sandwich beams with temperature-dependent material properties. Thin-Walled Struct..

[16] Zhang Z., Wang D., Yao L., Gu Z., Ke L., Xiao J. (2024). Asymptotic solutions for heat transfer and stresses in functionally graded porous sandwich pipes subjected to nonuniform pressures and thermal loads. Thin-Walled Struct..

[17] Tomczyk B., Gołąbczak M. (2020). Tolerance and asymptotic modelling of dynamic thermoelasticity problems for thin micro-periodic cylindrical shells. Meccanica.

[18] Tomczyk B., Gołąbczak M., Litawska A., Gołąbczak A. (2022). Mathematical modelling of thermoelasticity problems for thin periodic cylindrical shells. Contin. Mech. Thermodyn..

[19] Tomczyk B., Bagdasaryan V., Gołąbczak M., Litawska A. (2013). A new combined asymptotic tolerance model of thermoelasticity problems for thin biperiodic cylindrical shells. Compos. Struct..

[20] Ostrowski P., Jędrysiak J. (2021). Dependence of temperature fluctuations on randomized material properties in two-component periodic laminate. Compos. Struct..

[21] Jędrysiak J. (2000). On stability of thin periodic plates. Eur. J. Mech. A Solids.

[22] Jędrysiak J. (2007). The tolerance averaging model of dynamic stability of thin plates with one directional periodic structure. Thin-Walled Struct..

[23] Jędrysiak J., Kaźmierczak-Sobińska M. (2025). Free Vibration Analysis of Thin Functionally Graded Plate Bands with Microstructure as a Function of Material Inhomogeneity Distribution and Boundary Conditions. Materials.

[24] Chen Y.R., Chen L.W., Wang C.C. (2006). Axisymmetric dynamic instability of rotating polar orthotropic sandwich annular plates with a constrained damping layer. Compos. Struct..

[25] Wang H.J., Chen L.W. (2004). Axisymmetric dynamic stability of rotating sandwich circular plates. J. Vib. Acoust..

[26] Pawlus D. (2011). Dynamic stability of three-layered annular plates with wavy forms of buckling. Acta Mech..

[27] Pawlus D. (2011). Solution to the problem of axisymmetric and asymmetric dynamic instability of three-layered annular plates. Thin-Walled Struct..

[28] Garg A., Chalak H.D., Li L., Belarbi M.O., Sahoo R., Mukhopadhyay T. (2022). Vibration and Buckling Analyses of Sandwich Plates Containing Functionally Graded Metal Foam Core. Acta Mech. Solida Sin..

[29] Wolmir C. (1972). Nonlinear Dynamic of Plates and Shells.

[30] Wolmir C. (1967). Stability of Deformed System.

[31] Pawlus D. (2014). Dynamic response of three-layered annular plate with imperfections. Stud. Geotech. Mech..

[32] Pawlus D. (2022). Static stability of composite annular plates with auxetic properties. Materials.

[33] Wojciech S. (1980). Numerical determination of critical temperatures for annular plates. Arch. Mech. Eng..

[34] Trombski M., Wojciech S. (1981). The cylindrically orthotropic annular plate subjected to time-dependent pressure acting in its plane. Arch. Mech. Eng..

[35] Pawlus D. (2018). Sensitivity of Composite Structure with Directional Properties of Annular Three-Layered Plate Mechanically and Thermally Loaded. Mech. Mech. Eng..

